# Reducing the Delay in the Diagnosis of Bipolar Disorder: A Qualitative Study

**DOI:** 10.1111/hex.70398

**Published:** 2025-08-22

**Authors:** Hoppe, S. Watson, N. Ahuja, L. Azim, A. Cipriani, E. Clark, J. Evans, T. Gergel, J. Gibson, J. Hall, T. Kabir, A. Mathias, N. Nixon, Prasad, L. Wall, C. Chew‐Graham

**Affiliations:** ^1^ School of Medicine Keele University Keele UK; ^2^ Translational and Clinical Research Institute Newcastle University Newcastle upon Tyne UK; ^3^ Regional Affective Disorders Service, Cumbria, Northumberland, Tyne and Wear NHS Trust Newcastle upon Tyne UK; ^4^ Cumbria, Northumberland, Tyne and Wear NHS Trust Newcastle upon Tyne UK; ^5^ University of Oxford Oxford UK; ^6^ Oxford Precision Psychiatry Lab, NIHR Oxford Health Biomedical Research Centre Oxford UK; ^7^ NIHR Oxford Health Clinical Research Facility, Oxford Health NHS Foundation Trust, Warneford Hospital Oxford UK; ^8^ Cumbria, Northumberland, Tyne and Wear NHS Trust Newcastle University Newcastle upon Tyne UK; ^9^ Bristol University Bristol UK; ^10^ Bipolar London UK; ^11^ Division of Psychological Medicine and Clinical Neurosciences Cardiff University Cardiff UK; ^12^ Division of Psychiatry/Institute of Mental Health University College London London UK; ^13^ Centre for Humanities and Health, King's College London London UK; ^14^ McPin Foundation London UK; ^15^ Newcastle University Newcastle upon Tyne UK; ^16^ Department of Psychiatry University of Oxford Oxford UK; ^17^ School of Medicine University of Nottingham Nottingham UK; ^18^ University of Nottingham Nottingham UK

**Keywords:** bipolar disorder, diagnosis, patient journey, primary care, secondary care

## Abstract

**Introduction:**

Patients living with bipolar disorder in the UK face, on average, a delay of 9.5 years from initial presentation of symptoms to confirmation of diagnosis. The aim of this qualitative study was to understand the challenges and facilitators involved in diagnosing individuals with BD from the perspectives of GPs and psychiatrists and how the delay in diagnosis of BD from the first presentation might be reduced.

**Methods:**

Semi‐structured interviews with clinicians (GPs and psychiatrists) were used to explore attitudes and perspectives towards diagnosing, managing, and accessing or delivering specialist opinion for BD within the current NHS systems and pathways. Thematic analysis was conducted.

**Results:**

GPs report a lack of confidence in identifying BD due to limited understanding of the condition, resources, and lack of continuity of care. Both primary and secondary care clinicians expressed frustrations with the referral pathway in relation to high thresholds for secondary care acceptance and long waiting times for assessments.

Clinicians suggest that further education and training in primary care supported by psychometric tools and mood diaries to improve identification of BD. Clinicians also advocated for enhanced communication and collaboration between primary and secondary care to streamline and reduce delays in the diagnostic process.

**Conclusion:**

We suggest a number of strategies which could reduce the harmful delay in diagnosis of bipolar.

**Patient or Public Contribution:**

A Lived Experience Advisory Panel (LEAP) was convened with the support of the McPin Foundation. LEAP members have contributed towards the development of public‐facing documents, including the topic guides, qualitative data analysis and dissemination of findings.

## 1 Introduction

Bipolar disorder (BD) is a lifelong, recurring illness, based on a current or previous episode of mania or hypomania [1, 2]. BD may reduce life expectancy by 10–20 years [3, 4] caused by comorbid physical health problems (e.g. cardiometabolic diseases) and suicide [3, 5, 6]. With a lifetime prevalence of 2% [7, 8, 9], it is estimated that 40 million people worldwide currently live with bipolar disorder (BD), with 1.3 million living in the United Kingdom [10, 11]. It is estimated that treatment costs the United Kingdom (UK) £5.2 billion ($7 billion) per year (with direct costs of £324 million ($430 million) to the National Health Service (NHS) [12, 13].

The National Institute for Health and Care Excellence (NICE) outlines diagnostic processes that begin in primary care: the General Practitioner (GP) should screen for current or historical episodes of mania or hypomania, and if indicated, refer to secondary care for a specialist mental health assessment and formal diagnosis [14]. A complete psychiatric assessment is needed in specialist care to confirm or refute a diagnosis of BD before treatment plans, including medication and psychological intervention, are commenced. If an individual with BD responds to treatment, they are usually discharged to primary care for prescribing (under a shared care agreement), monitoring and ongoing care [14]. The Quality and Outcome Framework (QOF) contract in primary care suggests that patients on the Severe Mental Illness (SMI) register (which includes BD) should receive an annual physical health check, with additional monitoring for patients on lithium [15].

On average, 60% of people diagnosed with BD present with symptoms before age 21 years [16, 17]. In the UK, however, the recent Bipolar UK Commission of people living with BD found an average delay of 9.5 years between early symptoms, which might be suggestive of BD, and diagnosis/starting appropriate treatment [18]. Delays can severely affect an individual's psychological, social and occupational well‐being, contributing to decreased quality of life [19], and increased mortality rate [19], and healthcare costs [20, 21, 22]. If left untreated, individuals may face inappropriate or inadequate treatment, leading to escalated and more frequent manic episodes, increased rates of suicidal ideation, and hospital visits [20, 23, 24].

According to one NHS survey, from 2014, 56% of people living with BD had not received a formal diagnosis, and almost two‐thirds of people with a diagnosis did not receive any bipolar‐specific treatment or support [25]. Therefore, the 2022 Bipolar Commission [26] has called for better education about BD for the public and primary care, reduction in diagnostic delay, improved access to specialist support and treatment, better continuity of care, and a review of current NICE guidance [6].

GPs and Psychiatrists play an integral role in the BD diagnostic pathway, with GPs recognising symptoms which may represent bipolar and referring to specialist care. Psychiatrists are responsible for making the diagnosis of bipolar and initiating treatment. Therefore, the aim of this qualitative study was to understand the challenges and facilitators involved in diagnosing individuals with BD from the perspectives of GPs and psychiatrists and how the delay in diagnosis of BD from the first presentation might be reduced.

## 2 Materials and Methods

This nested qualitative study was part of a trial funded by the UK National Institute for Health and Care Research; Aripiprazole/sertraline combination: clinical and cost‐effectiveness in comparison with quetiapine for the treatment of bipolar depression (ASCEnD) [27].

### 2.1 Patient and Public Involvement and Engagement

Patient and Public Involvement and Engagement (PPIE) play a key role in the ASCEnD clinical trial and nested qualitative study. A Lived Experience Advisory Panel (LEAP) was convened with the support of the McPin Foundation [28]. The panel consisted of 10 members recruited to ensure diversity in sex, age, ethnicity and background. LEAP members contributed towards the development of public‐facing trial documents (e.g. the protocol), topic guides, training videos, and the trial website, via quarterly meetings. PPIE Co‐Investigators (JG and TK) contributed to monthly Trial Management Group (TMG) meetings, qualitative data analysis and dissemination activities [27].

### 2.2 Recruitment of Clinicians

Psychiatrists and GPs were invited to participate in a semi‐structured interview using purposive sampling to ensure that clinicians were either involved in the ASCEnD trial (and were on the trial delegation log) or who had direct experience of working with patients with bipolar disorder (recruited through professional networks, social media and snowball sampling). Psychiatrists acting as principal investigators (PIs) on the ASCEnD trial were not invited to participate to minimise conflicting interests and adversely affecting data sampling and interpretation. GPs were recruited using recruitment leaflets circulated via email through professional networks and social media (snowball sampling was also used) [27]. To ensure diversity, clinicians were sampled based on the range of trial sites, gender, age and ethnicity.

Before interview, clinicians were given a study information sheet, reminding them of their anonymity and made aware of their right to withdraw. Verbal consent was obtained at the beginning of each recorded interview. Since GPs are self‐employed, they were reimbursed for their time according to British Medical Association (BMA) rates [29].

### 2.3 Data Collection

First author (IH), a female psychology research associate with qualitative research experience from Keele University, conducted the semi‐structured interviews.

The researcher had no pre‐existing relationship with the clinicians and was transparent about the purpose of the research. The interviewer took a constructivist approach (attempting to understand experiences from the perspective of interviewees) while striving to remain engaged and open to the development of new ideas and themes [30]. The interviewer sought to respect varying views and ideas from clinicians' independent social, cultural, political, and ideological perspectives.

Interviews took place between June and February 2025 on a one‐to‐one basis either online or by telephone. Interviews were guided by topic guides for GPs and psychiatrists (Appendix 1 and 2) which included open‐ended questions and prompts. Topic guides were developed collaboratively with input from the qualitative team and LEAP, seeking to explore attitudes and perspectives towards diagnosing, managing, and accessing or delivering specialist opinion for BD within the current NHS systems and pathways. As data generation and analysis occurred, topic guides were modified iteratively with the input of the LEAP. Data collection continued until data saturation (or ‘theoretical sufficiency’) was achieved in both data sets, where the researcher can confidently support their interpretations [31, 32, 33].

### 2.4 Data Analysis

Interviews were digitally recorded capturing audio and/or visual and transcribed verbatim by The Transcription Company (UK). Transcripts, including demographic data, were checked for accuracy and anonymised with a unique participant identifier number. Transcripts were not returned to clinicians for feedback, comment, or correction. Field notes were made during the interview and supported analysis, performed by members of the qualitative research team using Glaser and Strauss' method of constant comparison [34]. This involved an inductive approach using thematic analysis by looking for connections within and across interviews, highlighting consistencies and variation. Thematic analysis was followed by framework analysis based on the Theoretical Framework of Acceptability across the data sets [33]. Analysis was carried out in collaboration with six members of the LEAP and two other researchers with backgrounds in qualitative research, using research software NVivo (version 11).

## 3 Results

Thirty‐three interviews were completed with 18 GPs and 15 psychiatrists. Clinician characteristics are reported in Table 1; 15 identified as male and 18 identified as female. There were no repeat interviews, or clinician withdrawals of consent or data from the study.

**Table 1:** Clinician demographics.

ID No.	Employment	Region	Age range (years)	Ethnicity
1	GP	West Midlands	60–70	White British
2	GP	North‐West	40–50	White British
3	GP	West Midlands	30–40	Black British
4	Psychiatrist	North‐West	30–40	Asian British
5	Psychiatrist	North‐West	Not stated	White British
6	Psychiatrist	North‐East	30–40	White British
7	Psychiatrist	North‐West	30–40	White non‐British
8	GP	South‐East	40–50	Asian British
9	GP	South‐East	Not stated	White non‐British
10	Psychiatrist	South‐East	30–40	White non‐British
11	GP	Yorkshire and the Humber	40‐–50	White British
12	GP	North‐West	na	White British
13	Psychiatrist	South‐East	40–50	White British
14	GP	London	30–40	Asian British
15	Psychiatrist	South‐East	Not stated	White non‐British
16	Psychiatrist	South‐West	30–40	White British
17	Psychiatrist	East‐Midlands	Not stated	Asian Indian
18	GP	North‐West	40–50	White British
19	GP	Yorkshire and the Humber	50–60	White British
20	GP	East Midlands	30–40	White British
21	Psychiatrist	South‐West	Not stated	Asian British
22	Psychiatrist	East Midlands	50–60	White Irish
23	GP	North‐West	30–40	White British
24	GP	North‐West	40–50	White British
25	GP	North‐West	40–50	Asian British
26	GP	North‐West	50–60	White British
27	GP	London	30–40	White British
28	Psychiatrist	North‐East	50–60	Asian Indian
29	GP	London	40–50	Asian British
30	Psychiatrist	West‐Midlands	30–40	Asian Indian
31	GP	North‐West	30–40	Asian British
32	Psychiatrist	London	50–60	White British
33	Psychiatrist	North‐West	30–40	White British

Each interview was conducted for 40 min on average with a range of 25 to 60 min.

The findings are presented under two themes: challenges and facilitators involved in reducing diagnostic delay, establishing a diagnosis and commencing treatment for BD. Data extracts are included to illustrate the themes and sub‐themes and annotated with clinician identifiers (GP or Psych and clinician number).

### 3.1 Challenges in the Diagnosis of BD

#### 3.1.1 Confidence and Competence

Some GPs described a lack of awareness of BD and reported they would not often consider this as a diagnostic possibility during consultations with people with mental health difficulties:

I think there is a lack of expertise, at least from my side. I don't know if other GPs would feel differently.

(GP9)

It wouldn't necessarily be something that would be super‐high on my radar to ask about periods of mania I've got to confess unless you started to get clues from the presentation or something that the patient specifically said themselves.

(GP19)

GPs reported varying confidence in identifying symptoms of BD; with GPs who were more experienced tending to report a greater awareness of BD and more confidence in considering this in consultations:

So, I would be pretty confident, as a senior doctor who's done lots of mental health, seen lots of mental, that he [the patient] has elements of bipolar.

(GP1)

Clinicians suggested that this could lead to an under‐diagnosis of BD, with service users being misdiagnosed and treated for conditions such as Depression or Emotionally Unstable Personality Disorder/Complex Emotional Needs:

So, I do feel there is a good chance, not only in my practice specifically, but all over, that this [BD] might be getting missed at times or that it gets misdiagnosed as depression, and we start to treat them as depression.

(GP9)

I mean, the big thing I always find difficult is discerning it from sort of personality disorder.

(GP12)

Some psychiatrists reported examples where they felt that GPs had misdiagnosed patients and attributed other conditions such as Posttraumatic Stress Disorder or depression as BD:

In my experience, I have come across patients who are being given the diagnosis of depression for a longer period, and then the hypomanic episode is not looked at, and then we have to sit down and go back to their past history and make the diagnosis [of BD].

(Psych21)

We used to get a lot of referrals from the GP saying that this patient is experiencing manic episode…but some of the time it is not a manic episode…So the patients who have been referred to us with the diagnosis of bipolar affective disorder, are not bipolar affective disorder themselves, most of them had personality problems, complex PTSD and yeah, most of them had complex PTSD.

(Psych17)

Lack of continuity of care across primary and secondary care was cited by some GPs as a reason for not considering a diagnosis of BD:

I think one of the problems with primary care at the moment is this lack of continuity of care. So if you're seeing different GPs all the time they're not going to see you in those different phases of your illness.

(GP24)

Many GPs highlighted pressures in primary care due to under‐resourcing and staffing challenges as a barrier to diagnosis:

I think everyone is so stretched, though, that they will do their level best, but it is a really stretched environment.

(GP8)

This time pressure was recognised by psychiatrists:

I think GPs are so busy at the moment, they are so pressurised with all their work that they may be not be able to give the time to their patients with bipolar.

(Psych13)

GPs expressed reluctance to prescribe antipsychotics and mood stabilisers without secondary care input:

Because if someone is kind of actively manic or hypomanic you might need to medicate them, in which case GPs aren't in the position to know what kind of antipsychotics or mood stabilisers they need to initiate or try…So I think GPs might have an underlying suspicion, but they are not comfortable or really have enough guidance for initiating medication.

(GP14)

#### 3.1.2 Where Does Responsibility for Diagnosis Lie?

All psychiatrists who were interviewed recognised that the diagnosis should ultimately be made in secondary care:

I don't think it's fair to expect a GP to make that diagnosis because they haven't had the specialist training in psychiatry that we have had.

(Psych5)

Likewise, the majority of GPs acknowledged the requirement for a BD diagnosis to be made in secondary care but described frustrations with the referral process due to rejection of referrals by specialist care, or, when accepted, long waiting times for assessment:

We might say rejected. Our secondary care colleagues might say redirected elsewhere…I mean I've more or less given up referring frankly. And when I have these informal discussions with other GPs, I think they're in the same situation, so probably the biggest barrier at the moment is our perception of how our referrals are going to be treated.

(GP26)

And so, you know, and so the fact that they have to wait 3 months just isn't good enough really because I mean these people are struggling day to day, hour to hour with their mental health for that amount of time which must be incredibly tiring for them, you know, even if they're not suicidal or if they're manic.

(GP20)

Likewise, Psychiatrists reflected on the referral process, acknowledging high thresholds for accepting referrals in secondary care:

The criteria are really quite high to get into a service and I think in those situations I can certainly understand why a GP might think they're not able to access that specialist knowledge easily. (Psych5)

The other issue is the community treatment team… they don't accept anybody unless you're about to jump from a cliff.

(Psych7)

Furthermore, many psychiatrists recognised that high referral thresholds and tensions between primary and secondary care can negatively impact the patient:

There is this division as in like GPs perceive secondary care as inaccessible in mental health and I think secondary care perceives GPs as lazy or I don't know what or not knowledgeable or doing inappropriate referrals or whatever and that ultimately impacts the patient I would say because it's the patient that's in the middle.

(Psych7)

Psychiatrists reflected on their own frustrations over the inflexibility of secondary care:

I also think whatever protocols that they've got of like if the patient doesn't turn up, is discharged, all of that stuff needs to be much more flexible, particularly when you are dealing with people who are mentally unwell, who may have loads of reasons why they are unable to leave their house or they have overslept or whatever, you need to adapt.

(Psych7)

Additional challenges in diagnosing BD included distinguishing between type I and type II BD, essential to determining the correct course of treatment for a patient. Some psychiatrists explained that even for trained psychiatrists, this can be challenging:

I think it's a difficult diagnosis to make. I think because bipolar 2 can be quite similar to other, like cyclothymia, or emotionally unstable personality disorder. I think that can be quite difficult. I think the diagnoses merge often change over time… But if you've seen somebody manic and somebody in a manic state, then that's easy to make that differential of which one it is. And then I think it is helpful because I think it does change the treatment plan slightly.

(Psych13)

I think it's paramount to make this distinction [between BP1 and 2]. It changes everything in terms of therapies, in terms of prognosis, in terms of management, in terms of therapies that we can use and in terms of even in risk for the patient.

(Psych10)

GPs described burdens around managing service user risk in the absence of specialist care involvement during periods of diagnostic uncertainty:

And then I think in primary care you feel a bit sort of abandoned because if someone says, ‘no it's not for us, and then you think ‘well who is it for, I'm not managing this’. So I can't – I feel like it's beyond me and so I've got no‐one else to turn to really.

(GP20)

And that's what I think isn't necessarily fair on GPs is that you're holding risk when you shouldn't be and if anything happened it feels like it's your responsibility, which is difficult.

(GP27)

#### 3.1.3 Working Around the System

Some GPs described feeling pressure to make exceptions for service users to help them receive a diagnosis, namely by justifying or exaggerating the risk when making referrals to secondary care.

You might over‐egg some things in the referral just because you feel like you have to. I've probably done that before…I think certainly emphasising things is increasingly something that you'd feel like you have to do if you want to get through some of these barriers that are in place.

(GP19)

This is what's bad, is I often sometimes slightly exaggerate. And I'm not lying, but you know, if a patient said to me like, ‘Yesterday I thought about doing this, I won't do it’ and I'm not actually worried about their risk imminently, but they have voiced some obviously really concerning things, I might not mention the fact that they've told me that they won't do it.

(GP27)

I've heard people say… if you use these buzz words, then sometimes that will expedite something.

(Psych33)

In some instances, GPs described taking other measures in attempt to meet referral thresholds:

If I'm really struggling to get a patient referred, I've escalated to the commissioner in a complaint, and then once it gets to the commissioner, they actually do accept the referral… the whole system is poor.

(GP23)

For service users with symptoms suggestive of BD, GPs reported commencing treatment without secondary care input:

I have myself personally effectively diagnosed a few people with what I think to be bipolar and commenced their management myself. I have done that because of a lack of confidence in the secondary care service.

(GP26)

### 3.2 Facilitators in the Diagnosis of BD

#### 3.2.1 Improving Confidence and Competence

Most clinicians suggested that education and training on BD for GPs could raise awareness, and improve the quality and rate of referrals to specialist care:

I think we should educate primary care clinicians a bit more…Because as I said, bipolar disorder overlaps with many mental health conditions. It can be personality disorder, it can be depression. And we don't always necessarily know, you know, like what is the case here and it's very easy to miss some of the things.

(GP9)

There could be some more education for primary care around presentation. [Yeah] Because if people were better prepared to understand, is this bipolar or is it not, maybe referrals would be taking place quicker?

(Psych13)

Many GPs described the use of mood diaries in accelerating the diagnosis of BD, a strategy echoed by secondary care:

I think the mood diary is really important. Whether that's something they sort of record or whether that's just something you go through in the consultation, and sort of what you're looking out for, so you know the elements to pick from that. But I think that's the biggest and most useful cue.

(GP12)

I suppose if people are coming to primary care and this is what they were maybe questioning or a GP was pondering, that process could be started potentially. They could be given mood diaries, they could be given some advice about keeping a record of their moods and symptoms over a period of time with a view to referring to us or already doing that. That potentially would speed it up.

(Psych6)

Some GPs discussed the use of psychometric tools for identification of possible BD symptoms and tracking their trajectory:

And I think in general practice it's always really helpful to have a formalised guide of what to look for and when and a process to go through that's a recognised process…I'm perhaps talking more about a specific, let's say a questionnaire‐based process that you repeat on several occasions that then you could track a score or track certain elements of that.

(GP24)

I don't know whether there's any scoring systems actually, I'm sure there probably are for someone with bipolar disease scoring system that I'm not aware of. But yeah, so that could probably help.

(GP20)

Psychiatrists agreed psychometric tools could support detection of BD in primary care:

I think you can also use some rating scales, if possible…if the GP is seeing the patient, if we could give a mood disorder question, or some tool which could pick up the subtle signs of bipolar affective disorder, like subclinical symptoms or those soft, soft signs of bipolar, it could be helpful for the assessing the next time.

(Psych17)

Clinicians suggested longer appointments and maintaining continuity of care could improve assessments for BD:

It would be much nicer…I think if you book a double appointment slot, so you have 20 minutes. That would be really helpful… I think GPs say this a lot, but time is the biggest resource we don't have…I think that'd be the one thing that would help us.

(GP12)

Also, from a patient's point of view, particularly for someone with bipolar, what they really value is having, say, a GP and the same with a consultant that they know long‐term but you don't get that anymore. It's very difficult for patients to achieve that. They're meeting locums or people who are on duty and that's a tremendous problem for a person with bipolar.

(Psych22)

#### 3.2.2 Primary‐Specialist Liaison

Most clinicians suggested enhanced communication across the primary‐secondary care interface might reduce delay in diagnosis, and improve service user experience and clinician collaboration and relationships:

I would really like to have a period of time in my diary set aside once a week where I can answer GP queries where it's kind of an open access available during that time for people within the sector that I cover so that if they do have concerns about a medication for someone whose not to our service or if they have had a referral rejected recently and they would like to kind of explore that in a bit more detail then we can explore that with them.

(Pscyh5)

Some way of building some more relationships with them [psychiatrists] would probably be helpful, and then you could potentially even be picking up the phone and having conversations with them on the phone and say, ‘look, this one's a bit off, could you see this one a bit sooner’

(GP12)

Moreover, clinicians suggested collaboration between primary and secondary care in the interim between referral and assessment could improve service user wellbeing during this period:

And what I'd like is a bridge in the interim. So for example if someone is really unwell medically and we know that something needs to be done, but they can be seen anytime soon, tell me what medication to start them on. Let's give it a go.

(GP14)

So I certainly don't think we should be asking GPs to take on all the work by any means, but I do think there needs to be a collaborative process and I think when that occurs that's the safest situation for everybody really.

(Psych5)

Many GPs noted that input from dedicated mental health practitioners and professionals contributes to the improvement of primary‐specialist collaboration:

And so that's why, if we do need to question it, going through our mental health practitioner nurse can be useful because she, well, she's great. She's a support for us with particular patients.

(GP11)

We have a Primary Care Mental Health Team that's got a psychologist and some other mental health workers, and they are designed for patients who fall between the cracks a little bit and get bounced back.

(GP19)

#### 3.2.3 Supporting Each Other

Clinicians highlighted mutual empathy and willingness to support colleagues across the primary‐specialist interface could facilitate earlier diagnosis of BD:

Yeah, I feel bad for GPs, to be honest. Because I feel like we should be doing more and often send them extremely long letters, which give them lots of things to do that I think that ultimately probably we should be doing if we are taking ultimate care of the patient.

(Psych16)

I think the biggest barrier is the fact that they're [psychiatrists] under‐resourced, so therefore they don't have, they're inundated, that supply‐demand that they can do is just completely mismatched. So, they're then overwhelmed…But I think that would be good. Some way of building some more relationships with them would probably be helpful.

(GP12)

## 4 Discussion

### 4.1 Summary of Findings

This qualitative study highlights the challenges in diagnosing BD and identifies potential facilitators that can be leveraged to accelerate the process, based on primary and secondary care clinician perspectives.

GPs report a lack of confidence in identifying BD due to limited understanding of the condition, resources (including time and staffing challenges), and patient continuity. These factors were believed to contribute to underdiagnosis and misdiagnosis of BD. Both primary and secondary care clinicians expressed frustrations with the referral pathway in relation to high thresholds for secondary care acceptance and long waiting times for assessments. GPs described a sense of responsibility for individuals presenting with possible BD symptoms, despite being unable to form a diagnosis themselves. In turn, GPs feel pressure to exaggerate patient risk to meet referral thresholds or commence treatment themselves despite lacking specialist psychiatric training.

To overcome these challenges, clinicians suggest that further education and training in primary care supported by psychometric tools and mood diaries to improve identification of BD. Clinicians also advocated for enhanced communication and collaboration between primary and secondary care as a means of streamlining the referral to diagnosis pipeline. Mutual clinician support could improve the quality of referrals, reduce waiting times and accelerate the time from onset of BD symptoms to diagnosis.

### 4.2 Strengths and Limitations

This study reports UK primary and secondary care clinician perspectives on factors associated with the well‐known delay between the first presentation of BD symptoms and diagnosis [18]. The involvement of LEAP and Bipolar UK was integral to this study, with LEAP contributing to the topic guides and data analysis.

Limitations include all clinicians being medics, future research should endeavour to gather insights from other mental health professionals, such as additional roles reimbursement scheme (ARRS) staff, mental health practitioners (MHPs), community psychiatric nurses (CPNs), health and wellbeing coaches, and social prescribing link workers. More GPs were recruited than psychiatrists, so primary care perspectives were over‐represented, although we achieved data saturation in both data‐sets. Furthermore, the majority of clinicians were based in the North of England; as such, the findings may not reflect the experiences of clinicians in other parts of the country and do not necessary apply to other healthcare systems outside the UK or non‐English nations.

### 4.3 Comparison With Existing Literature

This study provides insights into factors influencing delays in receiving a diagnosis of BD and contributes to the small pool of literature that currently explores this important concern. Many studies have sought to quantify the delay across the globe [18, 35, 36, 37, 38], but few have qualitatively explored the challenges and reasons behind this delay [39, 40]. The findings of this study align with the previous studies and provide a more detailed depiction of challenges and facilitators involved in diagnosing BD in the UK, specifically from GP and Psychiatry perspectives.

One study interviewing primary care clinicians in the US found similar barriers in diagnosing individuals with BD, including a lack of clinician understanding about BD, poor patient‐clinician continuity, long waiting times for secondary care, insufficient interface communication, and a lack of confidence in prescribing treatment without a confirmed diagnosis. Furthermore, their findings revealed comparable facilitators, including the use of psychometric tools and better collaboration with secondary care [39, 41].

Likewise, the challenges revealed by clinicians in this study are echoed in the UK's 2022 Bipolar Commission report, which cites a lack of clinician confidence leading to misdiagnosis amongst primary care clinicians, high referral thresholds and long wait times for secondary care assessments. Equally, several of the facilitators identified in this study are highlighted in the commission report's recommendations, including a need to improve confidence in primary care, provide continuity of care and reduce referral delays [40].

### 4.4 Implications for Research and/or Practice

There are several clear areas that need to be addressed to ensure that individuals living with BD receive a timely and accurate diagnosis and commence treatment as swiftly as possible. For example, in primary care, increased education, use of mood diaries and/or psychometric tools (e.g. Bipolar UK's recommended Mood Disorder questionnaire by Hirschfield and colleagues) (Appendix 3) [41, 42], longer appointments, and continuity of care may ensure that symptoms suggestive of BD are identified. Moreover, enhanced communication, streamlined referral processes, and collaboration between primary and secondary care may allow for a smoother referral process and responsive services. Harnessing these facilitators could reduce the delay in BD diagnosis and treatment in the UK; in doing so, individuals living with BD may benefit psychologically, socially and occupationally, enhancing quality of life [43]. Furthermore, early detection and diagnosis of BD could lead to improved prognosis and thereby reduce inpatient admissions and crisis team contacts, as well as associated healthcare costs. As healthcare systems continue to balance access with continuity, while facing unprecedented demands of adults seeking mental health assessments, research is essential to develop innovative treatment pathways (including digital pathways) [44]. Embedding research into clinical practice can help find a solution for real world practice. In line with existing initiatives, such as the Mental Health Mission [45] and clinical research hubs specifically dedicated to BD could facilitate access to care and generate evidence about best practices. This integrated approach would provide material support to the NHS and other healthcare systems, raising awareness and providing a responsive service to people with BD.

## Author Contributions

S.W. led grant application. C.C.‐G. and S.W. developed the study concept and design. I.H. performed the writing and revisions of the paper. I.H. and C.C.‐G. are responsible for data generation and initial analysis. J.G., T.K. and the ASCEnD lived experience group contributed to analysis of data. S.W., N.N., V.P. and N.A. contributed to data analysis. All authors read and approved the final paper.

## Ethics Statement

The study was approved by the Ethics and Health Research Authority (NHS Research Ethics Committee project reference number 23/NE/0132, IRAS number 1007468, and Research registry reference ISRCTN63917405).

## Conflicts of Interest

The views expressed are those of the authors and not necessarily those of the UK National Health Service, the NIHR, or the UK Department of Health and Social Care. During the course of this study Dr Prasad received salary funding via: King's College London from the National Institute for Health and Care Research (NIHR) academic clinical lecturer scheme; University of Nottingham as Associate Professor, via the NIHR senior clinical and practitioner research award (SCPRA) and Mental Health Mission; the NIHR East Midlands scholarship scheme (hosted by NHS Nottingham and Nottinghamshire and the University of Nottingham). Dr Prasad reports associations with King's College London and the University of Nottingham.

## Supporting information

Reducing the delay in the diagnosis of Bipolar Disorder ‐ Appendix.

## Data Availability

The data that support the findings of this study are available from the corresponding author upon reasonable request.
