# Supplementary material for: Reducing the Delay in the Diagnosis of Bipolar Disorder: A Qualitative Study
Source: Health Expect. 2025 Aug 22;28(4):e70398. doi: 10.1111/hex.70398 (PMC12371200; doi:10.1111/hex.70398)
Supplement: Supplementary file 1 — Reducing the delay in the diagnosis of Bipolar Disorder ‐ Appendix. [file HEX-28-e70398-s001.docx]

**Appendix 1 – Topic Guide GP**

**Background information**

Could you tell me some background information about yourself including:

- Sex, age, ethnic background (self-described)

**Interview topics to be covered**

1. Could you tell me about your practice? For example, the size (number of patients registered), it’s location (city/rural), if it is a training/teaching practice, and any particular characteristics (e.g. diversity/poverty/homeless residents/drug problems)?
   1. Do you know how many patients in your practice are on the SMI register (severe mental health illness register)? How many of these will have bipolar?
   2. Can you tell me anything about your QoF (the quality and outcomes framework) mental health scores?
2. Can you tell me about your practice in terms of diagnosis of bipolar disorder? [Prompt: for example, when people present with bipolar, but they do not have an established diagnosis of depression, do you also enquire about hypomanic episodes and/or consider bipolar a possibility?]
   1. Has this ever seemed appropriate, relevant, and your responsibility?
   2. What about people coming to the practice and telling you that they think they might have bipolar?
   3. Where do you think the diagnosis of bipolar should be made, by whom?
   4. What meaningful action(s) do you think patients can take to help speed up the diagnosis process? [Prompt: mood diaries]
   5. Do patients often have a family member/carer present at appointments? Are their insights helpful? How do you think we can work with them to help the diagnosis/management of patients with BD?
   6. How easy is it to refer a person you think has bipolar to specialist care?
   7. Can you tell me where your job ends and secondary care begins?
   8. When do you code a diagnosis of bipolar in a patient’s records?
   9. Do you think it is valuable for patients to receive a diagnosis of bipolar disorder? [Prompt: is it stigmatising or helpful? E.g. gives access to services]
3. In your opinion, what is the integration and communication between primary and secondary care like in your practice?
   1. Are there any barriers that exist between primary and secondary care?
   2. (if so) do you think this impacts diagnosis and/or care of patients with bipolar disorder?
   3. Could you tell me how this could possibly be improved? [prompts: communication, referral pathways, and discharge arrangements]
   4. [if not mentioned] How has the transformation of the care agenda affected the relationship between primary and secondary care?
4. Can you tell me about your management of people with an established diagnosis of bipolar?
   1. Do you provide ongoing care for people with bipolar disorder or are they typically in secondary /secondary care?
   2. Do you want to/feel the need to make treatment decisions?
   3. Where does, or where should, this responsibility lie between primary and secondary care?
   4. Do you ever feel the need to “bend the rules” in order to meet the needs of BD patients? [prompt: extend standard 10-minute appointments?]
5. How else do you think we could improve the diagnosis and management of people with bipolar disorder? [prompt: there is an average delayed of 9 years from first presentation to diagnosis]
6. What are the drug regimes that you have seen used in people with bipolar disorder? E.g. Antidepressants, Quetiapine, Olanzapine, Lithium etc.
   1. What are the implications for people with bipolar disorder (i.e. side-effects of medication; physical health problems, treatment burden, support – or lack of - from secondary care)
   2. How comfortable are you in monitoring drug regimes initiated by secondary care? {prompt: e.g. lithium]
7. Do you have any experience of prescribing Aripiprazole/Sertraline combination for people with bipolar disorder?
   1. (if so) Is this at the suggestion of a Psychiatrist? Would you need a shared care protocol to prescribe this combination?
   2. Would you be prepared to take over prescribing?
   3. What would be the impact on your or practice workload? (e.g. monitoring symptoms, side-effects, modifying doses and physical health monitoring)
8. Can you tell me how you came to be involved in the ASCEnD trial?
   1. Have you recruited any participants to the trial?
      1. Can you talk me through the process of recruitment?
         1. Did your practice send letters / text or ring people up to invite them to participate?
         2. Did you have any help from the RDN?
      2. Were there any challenges/difficulties in recruitment?
      3. Did you get any feedback from people who received invitations to participate in the trial?
      4. Are there any things that be done differently to improve recruitment?
   2. Were you involved in prescribing the trial medication?
      1. Can you tell me how that worked? Any difficulties?
   3. Were your experiences of the trial how you expected them to be?
      1. If yes, please explore; if no, why not?
9. Did you see/speak to any people in the ASCEnD trial who were participating in the study?
   1. How were you involved in physical health monitoring? What was it? (probe: weight, bloods, etc)
   2. How much did these people/this person feel they had been helped by the tablets they took?
   3. What were the side effects that this person may have complained about (if any)?
   4. Did you come into contact or any people who dropped out? Do you know why? Please outline their reasons.
10. What do you think will be the implications of the ASCEnD trial will be on your practice?
    1. For example… If Aripiprazole/Sertraline combination is more effective than Quetiapine – will this change your practice?
       1. If so, how will this affect resources (e.g. time, cost implications)?
    2. Conversely, if Quetiapine is more effective than the Aripiprazole/Sertraline combination, how will this affect your practice?
11. Do you have anything else you would like to add?

**End of interview**

Thank you for participating.

Arrange reimbursement.

Ask GP if they would be interested in receiving a summary of findings (and how we should communicate this).

**Close**

**Appendix 2 – Topic Guide Psychiatrist**

**Background information**

Could you tell me some background information about yourself including:

- Sex, age, ethnic background (self-described)

**Interview topics to be covered**

1. Could you tell me a little bit about your role? Years in your role? And any particular interests or expertise (e.g. diversity/poverty/homeless residents/drug problems)?
2. Can you tell me about your practice in terms of diagnosis of bipolar disorder? [Prompt: for example, when people present with bipolar, but they do not have an established diagnosis of depression, do you also enquire about hypomanic episodes and/or consider bipolar a possibility?]
   1. Whose responsibility is it to make a formal diagnosis of bipolar disorder?
   2. At what stage are you involved?
   3. Do you liaise with general practitioners over making a diagnosis?
   4. How do you feel about making a distinction between bipolar 1 and 2? How does it help?
   5. Do you think it is valuable for patients to receive a diagnosis of bipolar disorder? [Prompt: is it stigmatising or helpful? E.g. gives access to services]
   6. What meaningful action(s) do you think patients can take to help speed up the diagnosis process? [Prompt: mood diaries]
3. Can you tell me about your management of people with an established diagnosis of bipolar?
   1. Do you provide ongoing care for people with bipolar disorder? Do these people make a significant proportion of your caseload? How long do patients typically stay under your care?
   2. Do patients often have a family member/carer present at appointments? Are their insights helpful? How do you think we can work with them to help the diagnosis/management of patients with BD?
   3. Do you take on treatment decisions for people with bipolar disorder?
   4. Do you liaise with general practitioners over treatment (e.g. shared care agreement)?
   5. Where does, or where should, this responsibility lie between primary and secondary care?
4. In your option, what is the integration and communication between primary and secondary care like in your practice?
   1. Are there any barriers that exist between primary and secondary care?
   2. Do you find primary care ever exaggerates patient risk to get a referral through to secondary care?
   3. (if so) do you think this impacts diagnosis and/or care of patients with bipolar disorder?
   4. Could you tell me how this could possibly be improved? [prompts: communication, referral pathways, and discharge arrangements]
   5. [if not mentioned] How has the transformation of the care agenda affected the relationship between primary and secondary care?
5. How else do you think we could improve the diagnosis and management of people with bipolar disorder? [prompt: there is an average delayed of 9 years from first presentation to diagnosis]
   1. In your opinion, what Is your relationship and communication like with your patients?
   2. What typically strengthens this working relationship (e.g. access, responsiveness, etc.)
6. What are the drug regimes that you have seen used in people with bipolar disorder? E.g. antidepressants, Quetiapine, Olanzapine, lithium etc.
   1. What have the implications been for people with bipolar disorder (i.e. side-effects; physical health)
   2. Do you typically receive resistance from GP’s when it comes to monitoring drug regimens when the patient is being discharged back to primary care? [prompt: lithium]
7. Do you have any experience of prescribing Aripiprazole/Sertraline combination for people with bipolar disorder?
   1. Do you have any hopes or concerns over this combination?
   2. What is the impact on your or practice workload? (e.g. monitoring symptoms, side-effects, modifying doses and physical health monitoring)
8. Can you tell me how you came to be involved in the ASCEnD trial?
   1. Did you recruit any participants to the trial?
      1. Can you tell me how that worked and how successful that was? Any difficulties?
      2. WHY HAVEN’T YOU RECRUITED?
   2. Were you involved in prescribing the trial medication?
      1. Can you tell me how that worked? Any difficulties?
   3. Were your experiences of the trial how you expected them to be?
      1. If yes, please explore; if no, why not?
9. Did you see/speak to any people in the ASCEnD trial who were participating in the study?
   1. How were you involved in physical health monitoring? What was it? [prompt: weight, bloods, etc]
   2. How much did these people/this person feel they had been helped by the tablets they took?
   3. What were the side effects that this person may have complained about (if any)?
   4. Did you come into contact or any people who dropped out? Do you know why? Please outline their reasons.
10. What do you think will be the implications of the ASCEnD trial on your practice?
    1. For example…if Aripiprazole/Sertraline combination is more effective than Quetiapine – will this change your practice?
       1. If so, how will this affect resources (e.g. time, cost implications)?
    2. Conversely, if Quetiapine is more effective than the Aripiprazole/Sertraline combination, how will this affect your practice?
11. Do you have anything you wish to add?

**End of interview**

Thank you for participating.

Ask Psychiatrist if they would be interested in receiving a summary of findings (and how we should communicate this).

**Close**

**Appendix 3 – Mood Disorder Questionnaire**
